# The relationship between borderline symptoms and vantage perspective during autobiographical memory retrieval in a community sample

**DOI:** 10.1186/2051-6673-1-8

**Published:** 2014-06-11

**Authors:** Kris Van den Broeck, Jasmin Reza, Sabine Nelis, Laurence Claes, Guido Pieters, Filip Raes

**Affiliations:** Faculty of Psychology and Educational Sciences, University of Leuven, Tiensestraat 102, box 3712, 3000 Leuven, Belgium; University Psychiatric Centre KU Leuven Campus Kortenberg, Leuvensesteenweg 517, 3070 Kortenberg, Belgium

**Keywords:** Borderline personality disorder, Autobiographical memory, Vantage perspective, Imagery perspective, Self-discrepancy

## Abstract

**Background:**

Recent findings show that (previously) depressed and traumatised patients, compared to controls, make more frequently use of an observer perspective (as set against a field perspective) when retrieving memories. Because patients with borderline personality disorder (BPD) often report mood disturbances and past traumatic experiences, it would be plausible to expect that these patients too would retrieve higher proportions of observer memories. Therefore, and given the phenotypical variance of BPD, we examined whether vantage perspective during recall is associated with one or more BPD symptom clusters.

**Methods:**

A community sample consisting of 148 volunteers (66 males) completed the Autobiographical Memory Test, the Borderline Syndrome Index, and the Depression Scale of the Depression Anxiety Stress Scales.

**Results:**

Interpersonal and anxious-neurotic BPD features were associated with higher proportions of observer memories.

**Conclusions:**

The proportion of observer memories was not associated with the total number of BPD symptoms. Nevertheless, our data suggest the existence of substantial connections between perspective taking during recall on the one hand and interpersonal difficulties and anxious-neurotic symptoms on the other hand, especially following cues that tap into domains that are highly discrepant towards one’s actual self-concept.

## Background

“Autobiographical memory (AM) is the aspect of memory that is concerned with the recollection of personally experienced past events” [1], p. 122. It is believed to function as a kind of database of previous experiences on which we rely to find problem solving strategies when we experience difficulties [2], and it is thought to play an important role in the construction of our self-concept and in goal oriented behaviour [3]. It should not surprise, therefore, that problems with AM are found to be associated with emotional difficulties as major depressive disorder (MDD; [4]) and post-traumatic stress-disorder (PTSD; [4]). Recent findings [5, 6] reveal that depressed and traumatised patients differ from healthy controls with respect to the perspective they adopt while retrieving memories. Patients diagnosed with borderline personality disorder (BPD) frequently complain about depressive episodes and they often report past traumatic experiences [7]. Hence, it is reasonable to assume that people high on BPD complaints would show similar AM disturbances as depressed and traumatised patients. To our knowledge, vantage perspective has not been studied in relation to BPD symptoms hitherto. In this paper, we study the relationship between vantage perspective during retrieval and borderline complaints as measured in a community sample.

In general, most recollections are spontaneously remembered from one’s original point of view, as if one again 'sees’ the situation through one’s own eyes (i.e., field perspective) [8]. Nevertheless, some memories are remembered while 'seeing’ the situation as an observer might have seen it, also seeing oneself act in the situation (i.e., observer perspective or 'fly on the wall’ perspective). Field memories are generally experienced as more emotional and detailed, whereas in observer mode one is more likely to focus on the objective circumstances than on the affective elements [9–12] – but see also [13] for less straightforward findings and interpretations. Traumatised and (formerly) depressed patients tend to retrieve larger proportions of observer memories compared to controls (in traumatised patients: e.g., [9]; in currently depressed patients: e.g., [14]; in previously depressed patients: e.g., [15]).

According to Kuyken and Moulds [14], there are at least two possibly compatible accounts that give rise to higher proportions of observer memories in depressed and traumatised patients. First, Kuyken and Moulds [14] suggest that observer memories are used when one evaluates oneself, or when one needs to compare the actual self with a former or future self [16, 17]. Disappointments about goals that have not been attained and discrepancies between one’s actual self and one’s ideal self may lead to dejecting schemas about the self [18]. Such schemas may threaten the stability of the self. In order to prevent a reactive crash of the self, the Working Self, which is responsible for maintaining an integrated sense of self [3, 19, 20], specifically searches for information that is in line with the initial thoughts about the self. Hence, the organism is driven to evaluate oneself over and over, thereby using observer memories repeatedly. As expected, Kuyken and Moulds [14] found positive correlations between the number of observer memories on the one hand and levels of self-evaluation on the other (also see [21]).

Second, following McIsaac and Eich [11], Kuyken and Moulds [14] suggest that the recollection of more observer memories may function as a cognitive functional avoidance strategy: The adoption of an observer perspective then, at least on the short term, prevents one from getting overwhelmed by intense and possibly painful emotions associated with intrusive memories that are common in depression and PTSD. Indeed, both clinical observations and research data [5, 11, 22, 23] show that PTSD patients tend to report about their traumatic experiences while adopting an observer perspective, suggesting that the observer perspective may be adaptive in regulating, or at least dampening, one’s emotions. Furthermore, higher proportions of observer memories are found to be associated with outcomes on different avoidance measures (in depressed participants: e.g., [14, 24]; in traumatised participants: e.g., [11, 25]), again suggesting that the observer perspective may be an avoidance strategy.

Following the established associations between the use of an observer perspective during recall and MDD/PTSD, and given the presence of trauma and associated (painful) emotions, affect instability, and difficulties regarding the stability of the self in BPD patients (e.g., [26], but see also the BPD criteria in DSM-IV, [4]), we hypothesised that BPD features would be positively associated with the frequency of observer memories. Additionally, we explored the role of self-discrepancy in relation to vantage perspective during recall.

## Methods

### Participants

Hundred forty eight participants (66 males), all between 17 and 30 years of age (*M* = 21.34; *SD* = 3.22) participated and were recruited by the second author, Jasmin Reza, using her personal social network and its extensions (convenience sampling). The majority (60.2%) held a high school diploma, 11.5% held college level degrees, and 14.9% held master level degrees.

#### Instruments

**Autobiographical Memory Test (AMT,**[27]**; Dutch written minimal instructions version with adaptation towards the concept of discrepancy,**[28]**).** In a first phase participants were presented with 20 cue words. Clinicians had judged half of these cues to be high discrepant (HD) for depressed patients, whereas the other half was not (i.e. low discrepant, LD; for details, see [28]), but all cues had a positive valence. HD and LD cues were alternated, starting with a LD cue: *polite, happy, honest, enjoying, just, optimistic, respectful, energetic, sincere, satisfied, intelligent, pleasurable, neat, self-assured, sensitive, relaxed, grateful, successful, reliable*, and *active*. Respondents were instructed to write down autobiographical memories in response to each of the cues that were orally presented. Time limits were set at one minute per cue.

In a second phase participants were asked to go over their responses again and to judge vantage point of the retrieved memories by scoring each memory as 'p1’ (being a field memory or memory retrieved from a 1^st^ person perspective), or 'p3’ (being an observer memory or a memory retrieved from a 3^rd^ person perspective). Occasionally, respondents did not succeed to respond with a valid memory to a cue. These answers were coded as 'No Response’ by the experimenters afterwards. Respondents were also asked to rate their memories with respect to memory specificity, but these data go beyond the objective of this paper. Variable of interest is the total proportion of memories retrieved while using an observer perspective (%O). To compute %O, we only included the valid memories, excluding the answers coded as 'No Response’. Therefore, %O is the complement of the proportion of field memories. The written AMT has been used successfully previously to assess memory specificity (e.g., [29, 30]).

**Borderline Syndrome Index (BSI,**[31]**; Dutch translation,**[32]**).** The BSI consists of 52 items describing features and characteristics of BPD. Items are judged on presence, and should therefore be answered with 'yes’ or 'no’. Total scores range from 0 to 52, with higher scores reflecting greater levels of BPD pathology. Internal consistency in our sample was high, Cronbach’s alpha = .89, for the total score. A four factor model was revealed in the Dutch version [31]: Subscales measure Negative Self-Definition (NSD; 13 items, Cronbach’s alpha = .85), Difficult Interpersonal Relationships (DIR; 11 items, Cronbach’s alpha = .69), Failing Social Skills (FSS; 10 items, Cronbach’s alpha = .65), and Anxiety (ANX; 16 items, Cronbach’s alpha = .60). Convergent validity of the Dutch translation we used was found to be high, *r* = .75 (tested with the Diagnostic Interview for Borderline, [32]).

**Depression Anxiety Stress-Scales- Depression Scale (DASS21-D,**[33]**; Dutch version,**[34]**).** We were only interested in the Depression subscale (7 items, Cronbach’s alpha = .81 in the present study) of this 21-item self-report questionnaire. Respondents should score every item by indicating on a 4-point Likert scale to what extent the content of the item applied to them over the past week, ranging from 0 (*did not apply to me at all*) to 3 (*applied to me very much, or most of the time*). Higher scores indicate higher symptom severity. Psychometric properties were found to be good [34].

### Procedure

This study was part of a larger study. Following written informed consent participants were invited to complete a battery of measures, including the ones described above, in the order presented above. Data administration was organised individually. Except for the AMT, no time limits were set. Most participants finished the test battery within one hour. This study is approved by the Ethical Committee of KU Leuven.

## Results

### Sample characteristics

Table 1 includes the means, standard deviations, and ranges on all included variables. BPD complaints were limitedly reported (total BSI: *M* = 8.40, *SD* = 7.42, with a theoretical maximum score of 52), and depression severity (*M* = 3.32, *SD* = 3.56, with a theoretical maximum score of 21) scores were rather low. Thus, data are as expected in a community sample.

**Table 1:** **Descriptive statistics of the measures used**

	M	SD	Range
Autobiographical Memory Test (AMT)			
% O	27.77	.21	0.00 – 75.00
% O-HD	27.54	.23	0.00 – 80.00
% O-LD	27.78	.22	0.00 – 80.00
Total	4.11	.74	1.74 – 6.00
Borderline Syndrome Index (BSI)			
Negative Self-Definition (NSD)	1.59	2.47	.00 – 13.00
Difficult Interpersonal Relationships (DIR)	1.44	1.73	.00 – 10.00
Failing Social Skills (FSS)	2.31	1.97	.00 – 9.00
Anxiety (ANX)	2.77	2.42	.00 – 11.00
Total	8.40	7.42	.00 – 40.00
Depression scale of the Depression Anxiety Stress Scales (DASS21-D), total	3.32	3.56	0 – 17

%O = proportion memories retrieved during full AMT administration using an observer perspective; %O-HD = proportion memories retrieved using an observer perspective following high discrepant AMT cues; %O-LD = proportion memories retrieved using an observer perspective following low discrepant AMT cues.

### The relationship between vantage perspective during recall and borderline symptoms

Table 2 shows the correlations between the proportions of observer memories (in total as well as following high and low discrepant cues), and BPD symptom clusters as measured by the BSI. We controlled for depression severity (DASS21-D), to exclude that observed associations were solely due to shared associations with depressive symptoms, and for age, given that the proportion of observer memories was found to be significantly associated with participant’s age (see Table 3). The proportion of memories retrieved while using an observer perspective (%O) was not, as hypothesised, systematically associated with the total BSI score.

**Table 2:** **Correlations between proportions observer memories in total and following high and low discrepant cues, and borderline symptoms (BSI)**

		1	2	3	4	5	6	7	8
1.	% O	-	.90**	.88**	.04	.14	.17	.11	.14
2.	% O-HD	.90**	-	.60**	.06	.19*	.20*	.10	.16
3.	% O-LD	.88**	.57**	-	-.00	.06	.09	.09	.07
4.	BSI – NSI	.02	.05	-.02	-	.64**	.62**	.67**	.90**
5.	BSI – DIR	.14	.19*	.04	.52**	-	.51**	.49**	.77**
6.	BSI – FSS	.16	.20*	.08	.40**	.36**	-	.53**	.79**
7.	BSI – ANX	.11	.09	.10	.53**	.34**	.37**	-	.84**
8.	BSI – TOT	.15	.18	.07	.81**	.70**	.69**	.79**	-

Depression severity and age are partialled out in the correlations beneath the diagonal.

%O = proportion observer memories retrieved during full AMT administration; %O-HD = proportion observer memories following high discrepant cues; %O-LD = proportion observer memories following low discrepant cues; BSI-NSI = negative self-image; BSI-DIR = difficult interpersonal relationships; BSI-FSS = failing social skills; BSI-ANX = anxiety; BSI-TOT = total BSI-score.

*p < .05, **p < .01.

**Table 3:** **Correlations between depression severity (DASS-D), age, proportions observer memories in total and following high and low discrepant cues**

		2	3	4	5
1.	DASS-D	-.11	.04	.04	.02
2.	Age	-	-.26*	-.22*	-.25*
3.	% O		-	.90*	.88*
4.	% O-HD			-	.60*
5.	% O-LD				-

DASS-D = depression severity; %O = proportion observer memories retrieved during full AMT administration; %O-HD = proportion observer memories following high discrepant cues; %O-LD = proportion observer memories following low discrepant cues.

*p < .01.

### Further exploratory analyses

Table 1 includes the proportion of observer memories following HD and LD cues respectively. These proportions do not significantly differ, *t* = -.142, *p* = .888, nor do they differ from the proportion of observer memories retrieved irrespective of discrepancy (%O), *t* = -.278, *p* = .782, and *t* = .012, *p* = .991, for %O-HD and %O-LD versus %O, respectively.

We further explored the associations between the total proportion of observer memories and separate BPD symptom clusters, as well as the relationships between the proportions of observer memories retrieved following high (HD) and low (LD) discrepant cues and the BSI dimensions. Results are presented in Table 2. All reported *p*-values (except the ones discussed in the previous section) were corrected for multiple testing using the Benjamini-Hochberg’s method [35]. We found that observer perspective in the case of high discrepant cues (%O-HD) was prominently associated with Failing Social Skills (BSI-FSS), *r* = .20, *p <* .05, and with 'Difficult Interpersonal Relationships’ (BSI-DIR), *r* = .19, *p* < .05. These associations remained when controlling for age and depression severity, *r* = .20, and *r* = .19, respectively, both *p <* .05. No associations were found between BPD features and the proportion of observer memories following low discrepant cues (%O-LD). Given that these correlations are weak, we performed bootstrapping analyses in order to compute 95% confidence intervals around these correlations. Zero was never included in these intervals, 95% CI’s range from .020 to .339, and from .027 to .357, for the associations between %O-HD on the one hand and BSI-DIR and BSI-FSS on the other hand, respectively, both uncontrolled for age and depressive symptoms. When controlling for age and depressive symptoms, 95 CI’s range from .026 to .343, from .030 to .365, and from .018 to .333, for the associations between %O-HD on the one hand, and BSI-DIR, BSI-FSS, and BSI-total score on the other hand, respectively. Although these associations are weak, bootstrapping analyses suggest they are not trivial.

## Discussion

In this paper we investigated the relationships between vantage perspective of memories, and BPD features. Contrary to our predictions, no association was found between the total proportion of observer memories retrieved and the total number of BPD symptoms reported. Moreover, additional exploratory analyses on the association between cue discrepancy and vantage perspective revealed that equal proportions of observer memories were retrieved in response high and low discrepant cues. At first sight, these data suggest that BPD patients do not more often adopt an observer perspective during recall compared to healthy controls.

However, broadening our view to the role of cue discrepancy in relation to vantage perspective and BPD symptoms, we found that especially greater proportions of observer memories following *high discrepant cues* were associated with *'Difficult Interpersonal Relationships’* and *'Failing Social Skills’*, even when we controlled for depressive symptoms and age. These findings suggest that an observer perspective would be more common in those who are less socially skilled. However, taking a closer look into the FSS-subscale of the BSI, we discovered that these items rather refer to anxious-neurotic behaviours that are often thought to be typical for cluster C personality disorders [4]. Example items are, e.g., “I never accomplish as much as I could”, “I am afraid of anything new”, “It is hard for me to make decisions”, “I feel that I can not run my own life”, and “I feel uneasy in crowds, such as when I am shopping or at a movie”^a^. We therefore conclude that, besides difficult interpersonal relationships, cluster C type behaviours, rather than failing social skills, are associated with higher proportions of observer memories. This is in line with previous findings in (recurrent) depressed patients [6, 14, 15], who are known to hold higher dispositions on Neuroticism [36]. With respect to anxiety disorders, on the other hand, who also are considered to be more neurotic, the picture is less clear: Patients with different anxiety diagnosis differ in the perspective they adopt while retrieving memories [37]. In addition, since higher scores on FSS may also represent low self-esteem, our findings contradict the ones of Libby, Valenti, Pfent, and Eibach [38], who found no association between self-esteem and vantage perspective during recall.

Nevertheless, these findings potentially support the idea that high discrepant information may induce a tendency to compare, which is often associated with an observer mode [14]. According to another line of research, high discrepant cues also complicate the retrieval of memories on the content level [28, 39–42]. Traumatised as well as (previously) depressed patients show a tendency to retrieve categories of events (e.g., 'each time I went abroad for my job’) rather than memories referring to specific events that happened only once and did not take longer than one day (e.g., 'that one time in that Steak house in Toronto’). This is referred to as overgeneral memory (OGM; for an overview, see: [1, 43]). It is assumed that resources that are used during an intentional search process are taken away in favour of processes that aim to maintain a stable self-concept whenever the risk exists that painful memories will be reactivated. Moreover, it is assumed that the recall of overgeneral memories would be facilitated when respondents are explicitly asked to retrieve memories that are consistent with self-discrepant domains [42]. For instance, a depressed individual that is asked to retrieve a specific memory in response to the cue word 'happy’, will be more likely to refer to a category of events. Together with this line of reasoning, our results seem to suggest that a threatened self-concept thus might promote both the adoption of observer memories and the retrieval of overgeneral memories.

Yet, it should be noted that these correlations are weak, only explaining up to 4% of the other variable’s variance. Bootstrapping procedures, though, showed these associations are not trivial, but replications are recommended to heighten the validity of our findings.

Alternatively, broadening the view on imagery perspective to non-clinical subjects, the framework of Libby and Eibach [13] contradicts the above mentioned theories which both suggest that an observer perspective serves a dampening function. This model is built upon the widespread idea that the self is dual-faceted: One facet considers experiential awareness, the other conceptual knowledge. Whereas the first is fed by environmental features and concrete actions related to it, the latter consists of abstract meaning structures, defining the coherence of a self over time. According to Libby and Eibach [13], the perspective one adopts while retrieving memories, depends mainly on how one conceptualises the life event in relation to the facets of their self, and not, as hypothesised by the functional avoidance hypothesis, on the negative valence of the event for the individual. The adoption of a field perspective is assumed to address the experiential self, because this perspective evokes concrete features of a situation. An observer memory, on the other hand, would lead people to frame that event in a broader context, e.g., one’s self-beliefs, or in relation to other significant events. Difficult, self-discrepant memories are generally considered as highly relevant for one’s long-term self, and are more likely to be retrieved using an observer perspective. However, memories that emphasise the continuity in one’s self-concept may also be important in terms of the broader context of one’s life, and may therefore also been retrieved from an observer’s point of view. Moreover, the authors suggest that the adoption of an observer perspective also has the potential to intensify emotional reactions: “Third-person imagery increases emotional response relative to first-person imagery […] when the meaning of an event in the broader context of one’s life evokes a stronger emotional response than does the experience of the concrete details […]. However, when considering the meaning of an event in the broader context of one’s life evokes a weaker emotional response than does focusing on the concrete experience, third-person imagery should reduce emotional responses.” [13], p. 209-210. Unfortunately, our data do not allow to properly study these theoretical considerations, because we neglected to inventory the personal relevancy and/or the emotionality of the memories retrieved. We recommend that future studies would take these extra variables into account, in order to further distinguish between the possible functions of vantage perspective (dampening vs comparing vs meaning in life).

Our findings further suggest that, besides cluster C type behaviours, especially interpersonal BPD symptoms are associated with more observer memories. No clear cut explanations are available for these specific associations, although we speculate that empathy may be a key concept in attempts to clarify this relationship. Empathy, once defined as “the capacity to understand and respond to the unique affective experiences of another person” [44], p. 54 is generally assumed to be beneficial for interpersonal relationships. However, in relation to BPD, Krohn [45] identified the so-called 'borderline empathy paradox’. The paradox refers to the combination of seemingly enhanced empathic capabilities (see [46] for an overview) with impaired interpersonal functioning in BPD patients. Indeed, recent findings [47] suggest that BPD patients have increased levels of affective empathy ('sensing another’s feelings’ [48]: emotion recognition, emotional contagion) and decreased levels of cognitive empathy (perspective taking, theory of mind). More specifically, it is argued that BPD patients more intensely feel the other person’s feelings (i.e., higher levels of emotional contagion), and that they miss the higher-order cognitive empathy skills to cope with these intense feelings. Additionally, the intense feelings are presumable by the same mechanisms of emotional contagion reflected in the observer, resulting in mutual personal distress. We speculate that repetitive experiences of mutual personal distress would undermine the quality of the relationships. In addition, we further speculate that BPD patients, characterised by an unstable sense of self, probably eagerly tune in on all surrounded interpersonal stimuli they can find in order to create a sense of identity and safety. Their dysfunctional empathy system may be, at least in part, a maintaining element in their stable sense of self-instability. Of course, we are aware that a lot of speculations are formulated above that need further investigation. For instance, in relation to empathy, we talk about perspective taking *in general*, while in our and other studies vantage perspective *during recall* was questioned. Also, correlational research falls short in examining the causal relationships that are predicted above. Future research should try to reveal the true relations between these different kinds of imagery and all the hypothesised associations mentioned above.

Nevertheless, we underline that data were administered in a community sample, and not in a group of BPD patients. We need to be careful generalizing these (correlational) findings in our non-clinical sample to a clinical population, especially because the BSI-distributions are positively skewed and given that the associations found are weak, only explaining up to 4% of each other’s variance. Furthermore, we did not collect information on other aspects that can influence the AM characteristics of interest, such as traumatic experiences, posttraumatic symptoms, and medication use. Finally, cue discrepancy was not determined on a personal level, and with disregard of the clinical sample of interest (since the cues we used were judged to be discrepant for depressed patients, and not for BPD patients). Even so, we neglected to inventory other potentially interesting autobiographical memory variables (personal relevancy, emotionality, the extent to which a recalled memory is consistent with the presented cue), of which theorizing would benefit. Future research should therefore not only investigate whether it is possible to replicate the current findings in a clinical sample of BPD patients, but should also incorporate these extra variables to test the interesting hypotheses formulated above.

Interpersonal difficulties and problems with one’s self-concept are core features of BPD, causing a great deal of the burden of patients and their surroundings. Therefore, future studies should also explore the potential connections between (lack of aspects of) empathy, social impairments, vantage point, and dissociation. It has been suggested earlier [49] that dissociation is a highly defensive act in which all feelings are switched off. Rice and Rubin [50] suggested that vantage perspective would be more ecologically validly measured using a continuous scale, ranging from field to observer perspective. It could be hypothesised that dissociation is at the end of the scale, near observer perspective, since it can be considered the ultimate avoidance strategy. It thus would be interesting to find out how dissociative experiences relate to the quality of interpersonal experiences, and autobiographical mental imagery.

## Conclusions

To conclude then, and notwithstanding the above mentioned limitations, our findings are the first to our knowledge to suggest that vantage perspective during retrieval may be associated with BPD symptoms when cues activate domains that are highly discrepant towards the actual self.

## Endnotes

^a^Van den Wyngaert [51] acknowledges that this factor mainly consists of anxious-neurotic symptoms. However, he reasoned that all these symptoms could be thought as examples or consequences of inadequate social skills. Therefore, he chose to name the scale 'Failing Social Skills’.
